# Subspace mixed rational time-frequency multiwindow Gabor frames and their Gabor duals

**DOI:** 10.1186/s13660-018-1876-7

**Published:** 2018-10-11

**Authors:** Yan Zhang, Yun-Zhang Li

**Affiliations:** 1 School of Mathematics and Information Science, North Minzu University, Yinchuan, P.R. China; 2 College of Applied Sciences, Beijing University of Technology, Beijing, P.R. China

**Keywords:** 42C15, 42C40, Gabor frame, Mixed multiwindow Gabor frame, Dual, Oblique dual, Gabor dual

## Abstract

For a usual multiwindow Gabor system, all windows share common time-frequency shifts. A mixed multiwindow Gabor system is one of its generalizations, for which time-frequency shifts vary with the windows. This paper addresses subspace mixed multiwindow Gabor systems with rational time-frequency product lattices. It is a continuation of (Li and Zhang in Abstr. Appl. Anal. 2013:357242, 2013; Zhang and Li in J. Korean Math. Soc. 51:897–918, 2014). In (Li and Zhang in Abstr. Appl. Anal. 2013:357242, 2013) we dealt with discrete subspace mixed Gabor systems and in (Zhang and Li in J. Korean Math. Soc. 51:897–918, 2014) with $L^{2}(\mathbb{R})$ ones. In this paper, using a suitable Zak transform matrix method, we characterize subspace mixed multiwindow Gabor frames and their Gabor duals, obtain explicit expressions of Gabor duals, and characterize the uniqueness of Gabor duals. We also provide some examples, which show that there exist significant differences between mixed multiwindow Gabor frames and usual multiwindow Gabor frames.

## Introduction

Let $\mathcal{H}$ be a separable Hilbert space. An at most countable sequence $\{h_{i}\}_{i\in \mathcal{I}}$ in $\mathcal{H}$ is called a *frame* for $\mathcal{H}$ if there exist constants $0< A\leq B<\infty $ such that

1 $$ A\Vert f \Vert ^{2}\leq \sum _{i\in \mathcal{I}} \bigl\vert \langle f, h_{i}\rangle \bigr\vert ^{2}\leq B\Vert f \Vert ^{2}\quad \mbox{for } f\in\mathcal{H}, $$

where *A* and *B* are called *frame bounds*; it is called a *Bessel sequence* in $\mathcal{H}$ if the right-hand side inequality in (1) holds. In this case, *B* is called a *Bessel bound*. A frame for $\mathcal{H}$ is said to be a *Riesz basis* if it ceases to be a frame for $\mathcal{H}$ whenever an arbitrary element is removed. In this case, the frame bounds are also called *Riesz bounds*. The fundamentals of frames can be found in [3–6]. For $\lambda \in \mathbb{R}$, define the *modulation operator* $E_{\lambda }$ and *translation operator* $T_{\lambda }$ on $L^{2}(\mathbb{R})$ respectively by

$$ E_{\lambda }f(\cdot )=e^{2\pi i\lambda \cdot }f(\cdot )\quad \mbox{and} \quad T_{\lambda }f(\cdot )=f(\cdot -\lambda ) $$

for $f\in L^{2}(\mathbb{R})$. This paper addresses Gabor systems of the form

2 $$\begin{aligned} G(\mathbf{g},\mathbf{a},\mathbf{b})=\{E_{mb_{l}}T_{na_{l}}g_{l}:m,n \in \mathbb{Z}, 1\leq l\leq L\}, \end{aligned}$$

where *L* is a fixed positive integer, $\mathbf{g}=\{g_{l}: 1\leq l\leq L\}\subset L^{2}(\mathbb{R})$, $\mathbf{a}=(a_{1},a_{2},\ldots , a_{L})$, and $\mathbf{b}=(b_{1}, b_{2}, \ldots , b_{L})$ with $a_{l}, b_{l} >0$, $1\leq l\leq L$. We denote by $\mathcal{M}(\mathbf{g}, \mathbf{a}, \mathbf{b})$ the closed linear span of $G(\mathbf{g}, \mathbf{a}, \mathbf{b})$ in $L^{2}(\mathbb{R})$. A Gabor system $G(\mathbf{g}, \mathbf{a}, \mathbf{b})$ is called a *single-window Gabor system* if $L=1$; it is called a *mixed multiwindow Gabor system* if $L>1$ and $a_{l}$ (or $b_{l}$) with $1\leq l\leq L$ are not all the same; it is called a *multiwindow Gabor system* if $L>1$, $a_{1}=a_{2}=\cdots =a_{L}$, and $b_{1}=b_{2}=\cdots =b_{L}$. Similarly, $G(\mathbf{g}, \mathbf{a}, \mathbf{b})$ is called a *subspace single-window Gabor frame* if it is a frame for $\mathcal{M}(\mathbf{g}, \mathbf{a}, \mathbf{b})$ and $L=1$; it is called a *subspace mixed multiwindow Gabor frame* if it is a frame for $\mathcal{M}(\mathbf{g}, \mathbf{a}, \mathbf{b})$, $L>1$, and $a_{l}$ (or $b_{l}$) with $1\leq l\leq L$ are not all the same; it is called a *subspace multiwindow Gabor frame* if it is a frame for $\mathcal{M}(\mathbf{g}, \mathbf{a}, \mathbf{b})$, $L>1$, $a_{1}=a_{2}=\cdots =a_{L}$, and $b_{1}=b_{2}=\cdots =b_{L}$. In particular, when $\mathcal{M}(\mathbf{g}, \mathbf{a}, \mathbf{b})=L^{2}(\mathbb{R})$, these frames are usual frames, which have been extensively studied [7–12]. To distinguish from subspace frames, we call them *whole space frames*.

For a Bessel sequence $G(\mathbf{g}, \mathbf{a}, \mathbf{b})$ in $L^{2}(\mathbb{R})$, define the associated *synthesis operator* $\mathcal{T}_{\mathbf{g}}:l^{2}(\mathbb{Z}^{2},\mathbb{C}^{L})\rightarrow L^{2}(\mathbb{R})$ by

3 $$\begin{aligned} {\mathcal{T}}_{\mathbf{g}}c=\sum_{l=1}^{L} \sum_{m\in \mathbb{Z}} \sum_{n\in \mathbb{Z}}c_{l,m,n}E_{mb_{l}}T_{na_{l}}g_{l} \end{aligned}$$

for $c=(c_{1}, c_{2}, \ldots , c_{L})\in l^{2} (\mathbb{Z}^{2}, \mathbb{C}^{L})$. Then it is a bounded operator, and its adjoint operator $\mathcal{T}_{\mathbf{g}}^{*}$ (so-called *analysis operator*) is given by

$$ \mathcal{T}_{\mathbf{g}}^{*}f= \bigl( c_{1}(f), c_{2}(f), \ldots , c_{L}(f) \bigr) \quad \mbox{for }f\in L^{2}(\mathbb{R}), $$

where $c_{l}(f)= \{ \langle f, E_{mb_{l}}T_{na_{l}}g_{l} \rangle \} _{m, n \in \mathbb{Z}}$ for $1\leq l\leq L$. Similarly, for a Bessel sequence $G(\mathbf{h}, \mathbf{a}, \mathbf{b})$ in $L^{2}(\mathbb{R})$ with $\mathbf{h}=\{h_{1}, h_{2}, \ldots , h_{L}\}$, we associate it with $\mathcal{T}_{\mathbf{h}}$. Define $\mathcal{S}_{\mathbf{h},\mathbf{g}}=\mathcal{T}_{\mathbf{g}}{\mathcal{T}}_{\mathbf{h}}^{*}$, that is,

$$ {\mathcal{S}}_{\mathbf{h}, \mathbf{g}}f= \sum_{l=1}^{L} \sum\limits _{m\in \mathbb{Z}}\sum_{n\in \mathbb{Z}} \langle f, E_{mb_{l}}T_{na_{l}}h_{l}\rangle E_{mb_{l}}T_{na_{l}}g_{l} $$

for $f\in L^{2}(\mathbb{R})$. Let $G(\mathbf{g}, \mathbf{a}, \mathbf{b})$ be a frame for $\mathcal{M}(\mathbf{g}, \mathbf{a}, \mathbf{b})$, and let $G(\mathbf{h}, \mathbf{a}, \mathbf{b})$ be a Bessel sequence in $L^{2}(\mathbb{R})$. Then $G(\mathbf{h}, \mathbf{a}, \mathbf{b})$ is called an *oblique Gabor dual* for $G(\mathbf{g}, \mathbf{a}, \mathbf{b})$ if

$$ {\mathcal{S}}_{\mathbf{h},\mathbf{g}}f=f\quad \mbox{for }f\in {\mathcal{M}}(\mathbf{g}, \mathbf{a}, \mathbf{b}). $$

Here we do not require that $\mathbf{h}\subset {\mathcal{M}}(\mathbf{g}, \mathbf{a}, \mathbf{b})$. In particular, an oblique Gabor dual $G(\mathbf{h}, \mathbf{a}, \mathbf{b})$ for $G(\mathbf{g}, \mathbf{a}, \mathbf{b})$ is said to be a *Gabor dual of type* *I* for $G(\mathbf{g}, \mathbf{a}, \mathbf{b})$ if $\mathbf{h}\subset {\mathcal{M}}(\mathbf{g}, \mathbf{a}, \mathbf{b})$, and it is said to be a *Gabor dual of type II* for $G(\mathbf{g}, \mathbf{a}, \mathbf{b})$ if $\operatorname{range}(\mathcal{T}_{\mathbf{h}}^{*})\subset \operatorname{range}(\mathcal{T}_{\mathbf{g}}^{*})$. These notions are borrowed from [13] and [14]. Observe that a Gabor dual of type II is not required to be in $\mathcal{M}(\mathbf{g}, \mathbf{a}, \mathbf{b})$, but a moment containment relation is required.

For the whole space Gabor frames, single-window ones have been extensively studied in the past twenty years and more [4, 5, 7, 9, 15, 16]. Multiwindow frames were firstly studied by Zibulski and Zeevi [10] and Zeevi, Zibulski, and Porat [11]. By introduction of a Zak transform they developed a matrix (so-called Zibulski–Zeevi matrix) algebraic tool for multiwindow Gabor frames and applied it to image processing and computer vision. Since then, many researchers have studied multiwindow Gabor frames and related applications [2, 17–20]. It was also pointed out in [12] that the Zibulski–Zeevi matrix method is not very efficient for mixed Gabor frames. In [2], with the help of a new Zak transform matrix, different from the Zibulski–Zeevi matrix, Zhang and Li investigated mixed rational time-frequency multiwindow Gabor frames (Riesz bases and orthonormal bases) and their Gabor duals in $L^{2}(\mathbb{R})$. For subspace Gabor frames, single-window ones have been considered by several papers [1, 13, 21–26]. In [24, 26], and [27], a Zak transform matrix different from the Zibulski–Zeevi matrix was introduced and used effectively to study Gabor systems on periodic subsets of the real line, whereas the Zibulski–Zeevi matrix method does not work well for such Gabor systems. A variation of this method was applied to Gabor systems on discrete periodic sets [28, 29]. In [30], a density result for Gabor frames on periodic subsets of $\mathbb{R}^{d}$ is obtained via the Haar measure of the group generated by lattices. In [31], subspace multiwindow Gabor frames and their Gabor duals were characterized. All works mentioned, except [1] and [2], have not concerned real mixed multiwindow Gabor systems. Motivated by these observations, this paper is devoted to studying mixed multiwindow Gabor systems of the form (2). We work under the following assumptions:

### Assumption 1

*L* is a positive integer;

### Assumption 2

$b_{1}=b_{2}= \cdots =b_{L}=b$, and $a_{l}b=\frac{p_{l}}{q_{l}}$ with $p_{l}$ and $q_{l}$ being relatively prime positive integers for $1\leq l\leq L$.

We denote by $\mathbb{N}$ the set of positive integers. As it is pointed out in Remark 1.1 of [2], if $b_{1}, b_{2}, \ldots $ , $b_{L}$ in (2) are commensurable (there exist $b>0$, $\beta _{1}, \beta _{2}, \ldots $ , and $\beta _{L}\in \mathbb{N}$ such that $b=\beta _{l}b_{l}$ for $1\leq l\leq L$), then $G(\mathbf{g}, \mathbf{a}, \mathbf{b})$ is a frame (a Riesz basis, an orthonormal basis) for $\mathcal{M}(\mathbf{g}, \mathbf{a}, \mathbf{b})$ if and only if

$$ \bigl\{ e^{2\pi imb\cdot }g_{l}^{(\tau _{l})}(\cdot -na_{l}) :1\leq l\leq L, 0\le \tau _{l}\le {\beta _{l}}-1, m, n \in \mathbb{Z} \bigr\} $$

is a frame (a Riesz basis, an orthonormal basis) for $\mathcal{M}(\mathbf{g}, \mathbf{a}, \mathbf{b})$, where $g_{l}^{(\tau _{l})}(\cdot )=e^{2\pi i\tau _{l}b_{l}\cdot }g_{l}(\cdot )$. It is well known that $b_{1}, b_{2},\ldots, b_{L}$ are commensurable if they are all rational numbers or rational multiples of some fixed irrational number. We also remark that the restriction of “rational time-frequency” here is for using “finite-order” Zak transform matrix-valued functions. So Assumptions 1 and 2 are relatively general and reasonable to some extent.

Throughout this paper, *p* and *q* denote the least common multiple of $p_{l}$ and the greatest common divisor of $q_{l}$ with $1\leq l\leq L$, respectively. It is easy to check that *p* and *q* are relatively prime and that $\frac{p}{q}$ is the least common multiple of $\frac{p_{l}}{q_{l}}$ with $1\leq l\leq L$. So, for each $1\leq l\leq L$, there exists a unique $\lambda _{l}\in \mathbb{N}$ such that

4 $$ \frac{p}{q}=\frac{\lambda _{l}p_{l}}{q_{l}} . $$

This implies that $\lambda _{l}a_{l}=\frac{p}{b q}$ for $1\leq l\leq L$ by Assumption 2. We write

5 $$ a=\frac{p}{b q}\quad \mbox{and} \quad Q=q\sum {l=1}^{L} \lambda _{l}, $$

and we denote by $\mathbb{N}_{t}$ the set

$$ \mathbb{N}_{t}= \{ 0, 1, \ldots , t-1 \} $$

and by $I_{t}$ the $t\times t$ identity matrix for $t\in \mathbb{N}$. Hereinafter we use *I* to denote the identity matrix when we need not specify its size. Given a measurable set *S* in $\mathbb{R}$, a collection $\{S_{k}: k\in \mathbb{Z}\}$ of measurable sets in $\mathbb{R}$ is called a *partition* of *S* if

$$ \bigcup\limits _{k\in \mathbb{Z}}S_{k}=S\quad \mbox{and} \quad S_{k}\cap S_{k'} =\emptyset \quad \mbox{for }k\neq k' \mbox{ in }\mathbb{Z} $$

up to a set of measure zero. For $\lambda >0$ and measurable sets *S*, $S'\subset \mathbb{R}$, we say that *S* is $\lambda \mathbb{Z}$*-congruent* to $S'$ if there exists a partition $\{S_{k}: k\in \mathbb{Z}\}$ of *S* such that $\{S_{k}+\lambda k: k\in \mathbb{Z}\}$ is a partition of $S'$. In particular, only finitely many $S_{k}$ among $S_{k}$, $k\in \mathbb{Z}$, are nonempty if, in addition, both *S* and $S'$ are bounded. Obviously, $S'$ is also $\lambda \mathbb{Z}$-congruent to *S* if *S* is $\lambda \mathbb{Z}$-congruent to $S'$. So, in this case, we usually say that *S* and $S'$ are $\lambda \mathbb{Z}$-congruent. For *s*, $t\in \mathbb{N}$, we denote by $\mathcal{M}_{s, t}$ the set of all $s\times t$ complex matrices. Let $M\in {\mathcal{M}}_{s, t}$, which we consider as a linear mapping from $\mathbb{C}^{t}$ into $\mathbb{C}^{s}$, and define the mapping *M̃*: $\operatorname{ker}(M)^{\bot }\to {\mbox{ range}}(M)$ by $\tilde{M}x=Mx$ for $x\in (\operatorname{ker}(M))^{\bot }$. Then *M̃* is a bijection, and thus it has an inverse $(\tilde{M})^{-1}$. We extend $(\tilde{M})^{-1}$ to $M^{\dagger}$: $\mathbb{C}^{s} \to \mathbb{C}^{t}$ by defining

$$ M^{\dagger}(y+z)=(\tilde{M})^{-1}y,\quad y\in \operatorname{range}(M), z\in \bigl(\operatorname{range}(M) \bigr)^{\bot }. $$

The mapping $M^{\dagger}$ is called the *pseudo-inverse* of *M*.

The rest of this paper is organized as follows. In Sect. 2, using a suitable Zak transform matrix method, we characterize subspace mixed multiwindow Gabor frames, their Gabor duals of types I and II, and the uniqueness of Gabor duals and obtain explicit expressions of the Gabor duals. In Sect. 3, we give some examples and remarks. They show that there exist significant differences between mixed multiwindow Gabor frames and usual multiwindow Gabor frames. In particular, not every subspace mixed multiwindow Gabor frame $G(\mathbf{g}, \mathbf{a}, \mathbf{b})$ admits an oblique Gabor dual. So there should be many challenging problems in this direction.

## Frame and dual characterization

Let *L*, **a**, and **b** satisfy Assumptions 1 and 2. In this section, using a Zak transform matrix method, we characterize the Gabor systems $G(\mathbf{g}, \mathbf{a}, \mathbf{b})$ that are frames for $\mathcal{M}(\mathbf{g}, \mathbf{a}, \mathbf{b})$ and Gabor systems $G(\mathbf{h}, \mathbf{a}, \mathbf{b})$ that are duals of a frame $G(\mathbf{g}, \mathbf{a}, \mathbf{b})$ of types I and II. We also characterize the uniqueness of Gabor duals.

For $f \in L^{2}(\mathbb{R})$, define the *Zak transform* ${\mathcal{Z}}_{aq} f$ of *f* by

6 $$ ( {\mathcal{Z}}_{aq} f ) (t,v): =\sum _{\ell \in \mathbb{Z}}f(t+\ell aq) e^{2\pi i\ell v} $$

for a.e. $(t,v)\in \mathbb{R}^{2}$ and define

$$ {\mathbf{Z}}_{aq}f(t,v)= \left ( \textstyle\begin{array}{@{}c@{}} {\mathcal{Z}}_{aq}f(t,v) \\ \mathcal{Z}_{aq}f(t+\frac{1}{b}, v) \\ \vdots \\ \mathcal{Z}_{aq}f(t+\frac{p-1}{b}, v) \end{array}\displaystyle \right ) $$

for a.e. $(t,v)\in \mathbb{R}^{2}$. It is easy to check that the Zak transform has *quasi-periodicity*:

$$ ( {\mathcal{Z}}_{aq} f ) (t+kaq, v+\ell ) =e^{-2\pi ikv} ( { \mathcal{Z}}_{aq} f ) (t,v) $$

for $f\in L^{2}(\mathbb{R})$, $(k,\ell )\in {\mathbb{Z}}^{2}$ and a.e. $(t,v)\in \mathbb{E}^{2}$.

By Lemma 2.1 in [24], and by Lemma 2.1 in [2] we have the following:

### Lemma 2.1

(i) $\mathcal{Z}_{a q} ( E_{mb}T_{na q+x_{0}}f ) (t,v)=e^{2\pi imb t}e^{2\pi inv}{\mathcal{Z}}_{a q}f(t-x_{0}, v)$ *for* $f\in L^{2}(\mathbb{R})$, $x_{0}\in \mathbb{R}$ *and a*.*e*. $(t,v)\in \mathbb{R}^{2}$.
(ii) $\mathbf{Z}_{aq}$ *is a unitary operator from* $L^{2}(\mathbb{E})$ *onto* $L^{2}([0,\frac{1}{b})\times [0, 1), \mathbb{C}^{p})$, *and* $\mathcal{Z}_{aq}$ *is a unitary operator from* $L^{2}(\mathbb{E})$ *onto* $L^{2}( S\times [0,1))$ *for an arbitrary subset* *S* *of* $\mathbb{R}$ *thatis* $aq\mathbb{Z}$-*congruent to* $[0, aq)$.

### Definition 2.1

For $\mathbf{g}=\{g_{1}, g_{2}, \ldots , g_{L}\}\subset L^{2}(\mathbb{R})$, we associate it with a matrix-valued function $G(t,v):\mathbb{R}^{2} \rightarrow {\mathcal{M}}_{Q, p}$ by

7 $$\begin{aligned} G(t,v)=\left ( \textstyle\begin{array}{@{}c@{}} G_{1}(t,v) \\ G_{2}(t,v) \\ \vdots \\ G_{L}(t,v) \end{array}\displaystyle \right ) , \end{aligned}$$

where $G_{l}(t,v)$ is a block matrix of the form

8 $$ G_{l}(t,v)=\left ( \textstyle\begin{array}{@{}c@{}} {\mathcal{G}}_{l}(t,v) \\ \mathcal{G}_{l}(t-a_{l}, v) \\ \vdots \\ \mathcal{G}_{l}(t-(\lambda _{l}-1)a_{l} , v) \end{array}\displaystyle \right ) $$

with $\mathcal{G}_{l}(t,v):\mathbb{R}^{2} \rightarrow {\mathcal{M}}_{q, p}$ for $1\leq l\leq L$ and

$$\begin{aligned} {\mathcal{G}}_{l}(t,v)_{r,k}= \mathcal{Z}_{aq}g_{l} \biggl(t-ra+\frac{k}{b}, v \biggr) \end{aligned}$$

for $r\in \mathbb{N}_{q}$, $k\in \mathbb{N}_{p}$.

### Remark 2.1

By the quasi-periodicity of ${\mathcal{Z}}_{aq}$, for an arbitrary $f \in L^{2}(\mathbb{R})$, ${\mathcal{Z}}_{aq}f$ is uniquely determined by the values of $( {\mathcal{Z}}_{aq} f ) (\cdot , \cdot )$ on $S\times [0, 1)$ with *S* being a set $aq\mathbb{Z}$-congruent to $[0, aq)$. So, by Lemma 2.1(ii) an arbitrary function $F\in L^{2}(S\times [0, 1))$ determines a unique $f \in L^{2}(\mathbb{R})$ by

$$ ( {\mathcal{Z}}_{aq} f ) (t,v)= F(t,v)\quad \mbox{for } (t,v)\in S \times [0, 1). $$

Observe that $[0, \frac{1}{bq})-a\mathbb{N}_{q}+\frac{1}{b}\mathbb{N}_{p}$ is $aq\mathbb{Z}$-congruent to $[0, aq)$. It is easy to prove that if $a_{1}=a_{2}=\cdots =a_{L}$, then $Q=Lq$, and an arbitrary function ${M}(t,v): [0, \frac{1}{bq})\times [0, 1)\to {\mathcal{M}}_{Lq,p}$ with all entries in $L^{2}([0, \frac{1}{bq})\times [0, 1))$ determines a unique $\mathbf{g}=\{g_{1}, g_{2}, \ldots , g_{L}\}\subset L^{2}(\mathbb{R})$ by

$$ G(t,v)={M}(t,v)\quad \mbox{for a.e. } (t,v)\in \biggl[0, \frac{1}{bq}\biggr)\times [0, 1). $$

However, it is not the case if $a_{l}$, $1\le l\le L$, are not all the same. By an argument similar to that in [1], we have Example 2.1, which provides us with a counterexample. Therefore, we must be careful when we define **g** by a function ${M}(t,v): [0, \frac{1}{bq})\times [0, 1)\to {\mathcal{M}}_{Q,p}$ via

$$ G(t,v)={M}(t,v)\quad \mbox{for a.e. } (t,v)\in \biggl[0, \frac{1}{bq}\biggr)\times[0, 1) $$

if $a_{l}$, $1\le l\le L$, are not all the same.

### Example 2.1

Suppose $a_{l}$, $1\le l\le L$, are not all the same. Then there exists $1\le l\le L$ such that $\lambda _{l}>1$. We may as well assume that $\lambda _{1}>1$. Choose

$$ M(t,v)=\left ( \textstyle\begin{array}{@{}c@{}} M_{1}(t,v) \\ M_{2}(t,v) \\ \vdots \\ M_{L}(t,v) \end{array}\displaystyle \right ) , \qquad M_{l}(t,v)=\left ( \textstyle\begin{array}{@{}c@{}} {M}_{l,0}(t,v) \\ {M}_{l,1}(t,v) \\ \vdots \\ {M}_{l, \lambda _{l}-1}(t,v) \end{array}\displaystyle \right ) $$

for $1\leq l\leq L$ and $(t,v)\in [0, \frac{1}{bq})\times [0, 1)$ such that every entry of $M(t,v)$ belongs to $L^{2}([0, \frac{1}{bq})\times [0, 1))$ and

9 $$ {M}_{1,0}(t,v)\ne 0, \qquad {M}_{1,1}(t, v)= 0 \quad \mbox{for } (t,v)\in \biggl[0, \frac{1}{bq}\biggr)\times [0, 1). $$

Suppose there exists **g** such that $G(t,v)={M}(t,v)$ for a.e. $(t,v)\in [0, \frac{1}{bq})\times [0, 1)$. Then

10 $$\begin{aligned}& {\mathcal{G}}_{1}(t,v)={M}_{1,0}(t,v), \end{aligned}$$

11 $$\begin{aligned}& {\mathcal{G}}_{1}(t-a_{1}, v)={M}_{1,1}(t,v) \end{aligned}$$

for a.e. $(t,v)\in [0, \frac{1}{bq})\times [0, 1)$, where

$$ {\mathcal{G}}_{1}(t,v)_{r,k}= \mathcal{Z}_{aq}g_{1} \biggl(t-ra+\frac{k}{b}, v \biggr),\qquad {\mathcal{G}}_{1}(t-a_{1}, v)_{r,k}= \mathcal{Z}_{aq}g_{1} \biggl(t-a_{1}-ra+\frac{k}{b}, v \biggr) $$

for $(r, k)\in \mathbb{N}_{q}\times \mathbb{N}_{p}$. Since the sets $[0, \frac{1}{bq})-a\mathbb{N}_{q}+\frac{1}{b}\mathbb{N}_{p}$ and $[0, \frac{1}{bq}) -a\mathbb{N}_{q}+\frac{1}{b}\mathbb{N}_{p}-a_{1}$ are both $aq\mathbb{Z}$-congruent to $[0, aq)$, by the quasi-periodicity of ${\mathcal{Z}}_{aq}$ we have $\mathcal{Z}_{aq}g_{1}(t,v)\ne 0$ for $(t,v)\in E$ and some $E\subset [0, aq)\times [0, 1)$ with $\vert E \vert >0$ by (9), (10), whereas $\mathcal{Z}_{aq}g_{1}(t,v)=0$ for a.e. $(t,v)\in [0, aq)\times [0, 1)$ by (9) and (11). This is a contradiction.

Define the *Fourier transform* $\mathcal{F}$: $l^{2}(\mathbb{Z}^{2}) \rightarrow L^{2}([0, \frac{1}{b})\times [0, 1))$ by

$$ {\mathcal{F}}c(t,v)=\sqrt{b}\sum_{m\in \mathbb{Z}} \sum \limits _{n\in \mathbb{Z}}c_{m,n}e^{2\pi imbt}e^{2\pi inv} $$

for $c\in l^{2}(\mathbb{Z}^{2})$ and a.e. $(t,v)\in [0, \frac{1}{b})\times [0, 1)$, and define $\mathcal{J}$: $l^{2}(\mathbb{Z}^{2}, \mathbb{C}^{L}) \rightarrow L^{2}([0, \frac{1}{b})\times [0, 1), \mathbb{C}^{Q})$ by

$$ {\mathcal{J}}c(t,v)=\mathcal{C}(t,v) $$

for $c=(c_{1}, c_{2}, \ldots , c_{L})\in l^{2}(\mathbb{Z}^{2}, \mathbb{C}^{L})$, where

12 $$\begin{aligned}& {\mathcal{C}}(t,v)= \left ( \textstyle\begin{array}{@{}c@{}} {\mathcal{C}}_{1}(t,v) \\ \mathcal{C}_{2}(t,v)\\ \vdots \\ \mathcal{C}_{L}(t,v) \end{array}\displaystyle \right ) ,\qquad \mathcal{C}_{l}(t,v)=\left ( \textstyle\begin{array}{@{}c@{}} {\mathcal{C}}_{l}^{(0)}(t,v) \\ \mathcal{C}_{l}^{(1)}(t,v) \\ \vdots \\ \mathcal{C}_{l}^{(\lambda _{l}-1)} (t,v) \end{array}\displaystyle \right ) , \end{aligned}$$

13 $$\begin{aligned}& {\mathcal{C}}_{l}^{(\beta _{l})}(t,v)=\left ( \textstyle\begin{array}{@{}c@{}} \sum_{m\in \mathbb{Z}} \sum_{n\in \mathbb{Z}} c_{l,m,nq\lambda _{l}+\beta _{l}}e^{2 \pi imbt}e^{2\pi inv} \\ \sum_{m\in \mathbb{Z}} \sum_{n\in \mathbb{Z}} c_{l,m,(nq+1)\lambda _{l}+\beta _{l}}e^{2 \pi imbt}e^{2\pi inv} \\ \vdots \\ \sum_{m\in \mathbb{Z}} \sum_{n\in \mathbb{Z}} c_{l,m,(nq+q-1)\lambda _{l}+\beta _{l}}e^{2 \pi imbt}e^{2\pi inv} \end{array}\displaystyle \right ) \end{aligned}$$

for $1\leq l\leq L$ and $\beta _{l}\in \mathbb{N}_{\lambda _{l}}$. Similarly, for an arbitrary $d\in l^{2}( \mathbb{Z}^{2}, \mathbb{C}^{L})$, we associate it with $\mathcal{D}(t,v)$.

By a standard argument, we have

### Lemma 2.2

*The operators* $\mathcal{F}$ *and* $\sqrt{b}{\mathcal{J}}$ *are unitary operators from* $l^{2}(\mathbb{Z}^{2}, \mathbb{C}^{L})$ *onto* $L^{2}([0, \frac{1}{b})\times [0, 1))$ *and* $L^{2}([0,\frac{1}{b})\times [0, 1), \mathbb{C}^{Q})$, *respectively*.

By Lemmas 2.4 and 4.1 and by Remarks 2.6 and 2.7 in [31], we have following two lemmas.

### Lemma 2.3

*For* $\mathbf{g}=\{g_{1}, g_{2}, \ldots , g_{L}\}\subset L^{2}(\mathbb{R})$, *the following are equivalent*:

(i) $G(\mathbf{g}, \mathbf{a}, \mathbf{b})$ *is a Bessel sequence in* $L^{2}(\mathbb{R})$ *with Bessel bound B*.
(ii) $G(t,v)G^{*}(t,v) \leq bBI$ *for a*.*e*. $(t,v)\in [0, \frac{1}{bq})\times [0, 1)$.
(iii) $( G(t,v)G^{*}(t,v) ) ^{2} \leq bBG(t,v)G^{*}(t,v)$ *for a*.*e*. $(t,v)\in [0, \frac{1}{bq})\times [0, 1)$.
(iv) $G^{*}(t,v)G(t,v) \leq bBI$ *for a*.*e*. $(t,v)\in [0, \frac{1}{bq})\times [0, 1)$.
(v) $( G^{*}(t,v)G(t,v) ) ^{2} \leq bBG^{*}(t,v)G(t,v)$ *for a*.*e*. $(t,v)\in [0, \frac{1}{bq})\times [0, 1)$.
(vi) $\mathcal{Z}_{aq}g_{l}\in L^{\infty }(\mathbb{R}^{2})$ *for* $1\le l\le L$.

### Lemma 2.4

*For* $\mathbf{g}=\{g_{1}, g_{2}, \ldots , g_{L}\}\subset L^{2}(\mathbb{R})$, $G(\mathbf{g}, \mathbf{a}, \mathbf{b})$ *is complete in* $L^{2}(\mathbb{R})$ *if and only if*

$$ \operatorname{rank} \bigl(G(t,v) \bigr)=p\quad \textit{ffor a.e. } (t,v)\in \biggl[0, \frac{1}{bq}\biggr)\times [0, 1). $$

### Lemma 2.5

*For* $\mathbf{g}=\{g_{1}, g_{2}, \ldots , g_{L}\} \subset L^{2}(\mathbb{R})$, *we have*

(i) $$\begin{aligned}& \langle f, E_{mb}T_{na q+ra+\tau a_{l}}g_{l}\rangle \\& \quad = \int _{0}^{\frac{1}{b}} \int _{0}^{1} \bigl( \overline{ \mathcal{G}_{l}(t-\tau a_{l}, v)} {\mathbf{Z}}_{a q}f(t, v) \bigr) _{r}e^{-2\pi imb t} e^{-2\pi inv}\,dt\,dv \end{aligned}$$
  *for* $f\in L^{2}(\mathbb{R})$, $1\le l\le L$ *and* $(m, n, r, \tau )\in \mathbb{Z}\times \mathbb{Z}\times \mathbb{N}_{q}\times \mathbb{N}_{\lambda _{l}}$.
(ii) $G(t+\frac{n}{bq}, v)=e^{- 2\pi im_{n}v} {\mathcal{C}}_{n} G(t,v) \mathcal{D}_{n} $ *for* $n=k_{n}q+(m_{n}q-r_{n})p$ *with* $(k_{n},r_{n},m_{n})\in \mathbb{N}_{p}\times \mathbb{N}_{q}\times \mathbb{Z}$ *and a*.*e*. $(t,v)\in \mathbb{R}^{2}$, *where*
  $$\begin{aligned}& \mathcal{D}_{n}= \left ( \textstyle\begin{array}{@{}c@{\quad}c@{}} 0 & e^{-2 \pi iv}I_{k_{n}} \\ I_{p-k_{n}} & 0 \end{array}\displaystyle \right ), \end{aligned}$$
  $\mathcal{C}_{n}=\operatorname{diag}(\mathcal{C}_{1,n}(v), \mathcal{C}_{2,n}(v), \ldots , \mathcal{C}_{L,n}(v))$, $\mathcal{C}_{l,n}(v)$ *denotes the block matrix* (*with* $\lambda _{l}$ *blocks*) *of the form* $\operatorname{diag}({C}_{n}, {C}_{n}, \ldots , {C}_{n})$ *with*
  $$\begin{aligned}& C_{n}= \left ( \textstyle\begin{array}{@{}c@{\quad}c@{}} 0 & I_{q-r_{n}} \\ e^{2\pi iv}I_{r_{n}} & 0 \end{array}\displaystyle \right ). \end{aligned}$$
(iii) $\mathbf{Z}_{aq}{\mathcal{T}}_{\mathbf{g}}c(t,v)=\overline{G^{*}(t,v)} \mathcal{J}c(t,v)$ *for a*.*e*. $(t,v)\in \mathbb{R}^{2}$ *whenever* $c\in l_{0}(\mathbb{Z}^{2}, \mathbb{C}^{L})$ (*that is*, *c* *is finitely supported*) *or* $G(\mathbf{g}, \mathbf{a}, \mathbf{b})$ *is a Bessel sequence in* $L^{2}(\mathbb{R})$, *where* $\mathcal{T}_{\mathbf{g}}c$ *is as in* (3).
(iv) *If* $G(\mathbf{g}, \mathbf{a}, \mathbf{b})$ *is a Bessel sequence in* $L^{2}(\mathbb{R})$, *then*
  $$ \mathcal{J}\mathcal{T}_{\mathbf{g}}^{*}f(t,v)=\frac{1}{b}\overline{G(t,v)} {\mathbf{Z}}_{aq}f(t,v) $$
  *for* $f\in L^{2}(\mathbb{R})$ *and a*.*e*. $(t,v)\in \mathbb{R}^{2}$.

### Proof

(i), (ii), and (iii) are from Lemmas 2.2, 2.3, 2.5 in [2]. Next, we prove (iv). Write $d=\mathcal{T}_{\mathbf{g}}^{*}f=(d_{1}, d_{2}, \ldots , d_{L})$. By Lemma 2.1 we have

14 $$\begin{aligned}& d_{l,m,(nq+r)\lambda _{l}+\beta _{l}} \\& \quad =\sum_{k\in \mathbb{N}_{p}} \int _{0}^{\frac{1}{b}} \int _{0}^{1}{\mathcal{Z}}_{aq}f \biggl(t+ \frac{k}{b}, v \biggr)\overline{\mathcal{Z}_{aq}g_{l} \biggl(t-\beta _{l}a_{l}-ra+\frac{k}{b}, v \biggr)}e^{-2\pi imbt}e^{-2\pi inv} \,dt\,dv \\& \quad = \int _{0}^{\frac{1}{b}} \int _{0}^{1} \bigl( \overline{ \mathcal{G}_{l}(t-\beta _{l}a_{l}, v)} { \mathbf{Z}}_{aq}f(t,v) \bigr) _{r}e^{-2\pi imbt}e^{-2\pi inv}\,dt\,dv \end{aligned}$$

for $(r, \beta _{l})\in \mathbb{N}_{q}\times \mathbb{N}_{\lambda _{l}}$, $1 \leq l\leq L$. When $G(\mathbf{g}, \mathbf{a}, \mathbf{b})$ is a Bessel sequence, the integrand in (14) belongs to $L^{2}([0, \frac{1}{b})\times [0, 1))$ by Lemma 2.3(vi). It follows that

$$ {\mathcal{D}}^{(\beta _{l})}(t,v)=\frac{1}{b}\overline{ \mathcal{G}_{l}(t-\beta _{l}a_{l}, v)} { \mathbf{Z}}_{aq}f(t,v) $$

for a.e $(t,v)\in [0, \frac{1}{b})\times [0, 1)$. This leads to the lemma. □

### Remark 2.2

By Lemma 2.5(ii),

$$ \operatorname{rank} \biggl(G \biggl(t+\frac{n}{bq}, v \biggr) \biggr)= \operatorname{rank} \bigl(G(t,v) \bigr), $$

$$ G^{*} \biggl(t+\frac{n}{bq}, v \biggr)G \biggl(t+ \frac{n}{bq}, v \biggr)=\mathcal{D}_{n}^{*}G^{*}(t,v)G(t,v) \mathcal{D}_{n}, $$

$$ G \biggl(t+\frac{n}{bq}, v \biggr)G^{*} \biggl(t+ \frac{n}{bq}, v \biggr)=\mathcal{C}_{n}G(t,v)G^{*}(t,v) \mathcal{C}_{n}^{*} $$

for $(t,v)\in \mathbb{R}^{2}$ and $n\in \mathbb{Z}$. It follows that the range of $\operatorname{rank}(G(t,v))$ on $[0, \frac{1}{bq})\times [0, 1)$ is that on $\mathbb{R}^{2}$, and the spectrum properties of $G^{*}(t,v)G(t,v)$ and $G(t,v)G^{*}(t,v)$ on $[0, \frac{1}{bq})\times [0, 1)$ determine their spectrum properties on $\mathbb{R}^{2}$. For simplicity, all theorems further will be stated on $[0, \frac{1}{bq})\times [0, 1)$.

By an argument similar to Lemmas 27, 28 in [1] and Lemmas 3.3, 3.4 in [31], we have the following two lemmas.

### Lemma 2.6

*For* $\mathbf{g}=\{g_{1}, g_{2}, \ldots , g_{L}\}$, $\mathbf{h}=\{h_{1}, h_{2}, \ldots , h_{L}\}\subset L^{2}(\mathbb{R})$, *the following are equivalent*:

(i) $\mathbf{h}\subset {\mathcal{M}}(\mathbf{g}, \mathbf{a}, \mathbf{b})$.
(ii) *there exists a measurable function* $A:[0, \frac{1}{bq}) \times [0, 1) \rightarrow {\mathcal{M}}_{Q, Q}$ *such that* $H(t,v)=A(t,v)G(t,v)$ *for a*.*e*. $(t,v)\in [0, \frac{1}{bq})\times [0, 1)$.
(iii) *there exists a measurable function* $A:\mathbb{R}^{2} \rightarrow {\mathcal{M}}_{Q, Q}$ *such that* $H(t,v)=A(t,v)G(t,v)$ *for a*.*e*. $(t,v)\in \mathbb{R}^{2}$.

### Lemma 2.7

*Given* $\mathbf{g}=\{g_{1}, g_{2}, \ldots , g_{L}\} $, $\mathbf{h}=\{h_{1}, h_{2}, \ldots , h_{L}\}\subset L^{2}(\mathbb{R})$, *let* $G(\mathbf{g}, \mathbf{a}, \mathbf{b})$ *and* $G(\mathbf{h}, \mathbf{a}, \mathbf{b})$ *be Bessel sequences in* $L^{2}(\mathbb{R})$. *Then the following are equivalent*:

(i) $\operatorname{range}(\mathcal{T}_{\mathbf{h}}^{*})\subset\overline{\operatorname{range}(\mathcal{T}_{\mathbf{g}}^{*})}$.
(ii) *there exists a measurable function* $B:[0, \frac{1}{bq})\times [0, 1)\rightarrow {\mathcal{M}}_{p,p}$ *such that*
  $$ H(t,v)=G(t,v)B(t,v)\quad \textit{for a.e. } (t,v)\in \biggl[0, \frac{1}{bq}\biggr) \times [0, 1). $$
(iii) *there exists a measurable function* $B:\mathbb{R}^{2} \rightarrow {\mathcal{M}}_{p,p}$ *such that*
  $$ H(t,v)=G(t,v)B(t,v)\quad \textit{for a.e. } (t,v)\in \mathbb{R}^{2}. $$

### Lemma 2.8

*Given* $\mathbf{g}=\{g_{1}, g_{2}, \ldots , g_{L}\}$, $\mathbf{h}=\{h_{1}, h_{2}, \ldots , h_{L}\}\subset L^{2}(\mathbb{R})$, *let* $G(\mathbf{g}, \mathbf{a}, \mathbf{b})$ *and* $G(\mathbf{h}, \mathbf{a}, \mathbf{b})$ *be Bessel sequences in* $L^{2}(\mathbb{R})$. *Then*

15 $$\begin{aligned} {\mathbf{Z}}_{aq}(\mathcal{S}_{\mathbf{h},\mathbf{g}}f) (t,v)= \frac{1}{b}\overline{G^{*}(t,v) H(t,v)} {\mathbf{Z}}_{aq}f(t, v) \end{aligned}$$

*for* $f\in L^{2}(\mathbb{R})$ *and a*.*e*. $(t,v)\in \mathbb{R}^{2}$.

### Proof

Since $\mathcal{S}_{\mathbf{h},\mathbf{g}}=\mathcal{T}_{\mathbf{g}}{\mathcal{T}}_{\mathbf{h}}^{*}$, applying Lemma 2.5(iii), (iv) leads to the lemma. □

### Lemma 2.9

*Given* $\mathbf{g}=\{g_{1}, g_{2}, \ldots , g_{L}\} $, $\mathbf{h}=\{h_{1}, h_{2}, \ldots , h_{L}\}\subset L^{2}(\mathbb{R})$, *let* $G(\mathbf{g}, \mathbf{a}, \mathbf{b})$ *and* $G(\mathbf{h}, \mathbf{a}, \mathbf{b})$ *be Bessel sequences in* $L^{2}(\mathbb{R})$. *Then the following are equivalent*:

(i) $G(\mathbf{h}, \mathbf{a}, \mathbf{b})$ *is an oblique Gabor dual for* $G(\mathbf{g}, \mathbf{a}, \mathbf{b})$.
(ii) $G^{*}(t,v)=\frac{1}{b}G^{*}(t,v)H(t,v) G^{*}(t,v)$ *for a*.*e*. $(t,v)\in [0, \frac{1}{bq})\times [0, 1)$.
(iii) $G^{*}(t,v)=\frac{1}{b}G^{*}(t,v)H(t,v) G^{*}(t,v)$ *for a*.*e*. $(t,v)\in \mathbb{R}^{2}$.

### Proof

By Lemma 2.5(ii), (ii) and (iii) are equivalent. So, to prove the lemma, it suffices to prove the equivalence between (i) and the following equation:

16 $$\begin{aligned} G^{*}(t,v)=\frac{1}{b}G^{*}(t,v)H(t, v) G^{*}(t,v)\quad \mbox{for a.e. } (t,v)\in \biggl[0, \frac{1}{b}\biggr) \times [0, 1). \end{aligned}$$

Since $\operatorname{range}(\mathcal{T}_{\mathbf{g}})$ is dense in $\mathcal{M}(\mathbf{g}, \mathbf{a}, \mathbf{b})$, (i) holds if and only if

$$ {\mathcal{S}}_{\mathbf{h},\mathbf{g}}{\mathcal{T}}_{\mathbf{g}}c=\mathcal{T}_{\mathbf{g}}c \quad \mbox{for }c\in l^{2} \bigl(\mathbb{Z}^{2}, \mathbb{C}^{L} \bigr) $$

or, equivalently,

$$ \overline{G^{*}(t,v)} {\mathcal{J}}c(t,v)= \frac{1}{b} \overline{G^{*}(t,v)H(t,v) G^{*}(t,v)} {\mathcal{J}}c(t, v)\quad \mbox{for } c\in l^{2} \bigl(\mathbb{Z}^{2}, \mathbb{C}^{L} \bigr) $$

by Lemmas 2.1, 2.5, and 2.8, which is in turn equivalent to

17 $$\begin{aligned} G^{*}(t,v)d(t,v)=\frac{1}{b}G^{*}(t, v)H(t,v) G^{*}(t,v)d(t,v) \end{aligned}$$

for $d(t,v)\in L^{2}([0,\frac{1}{b})\times [0,1), \mathbb{C}^{Q})$ by Lemma 2.2. Obviously, (16) implies (17). Now suppose (17) holds. For arbitrary fixed $x\in \mathbb{C}^{Q}$, choose $d(t,v)$ as

$$ d(t,v)=x \quad \mbox{for } (t,v)\in \biggl[0,\frac{1}{b}\biggr)\times [0,1). $$

Then $d(t,v)\in L^{2}([0, \frac{1}{b})\times [0, 1), \mathbb{C}^{Q})$, and thus

$$ G^{*}(t,v)x=\frac{1}{b}G^{*}(t,v)H(t,v) G^{*}(t,v)x $$

for a.e. $(t,v)\in [0, \frac{1}{b})\times [0, 1)$ by (17). So (16) holds by the arbitrariness of *x*. The proof is completed. □

By the definition of pseudo-inverse, we have following two lemmas.

### Lemma 2.10

*For a* $d\times d$ *matrix* *A* *satisfying* $A^{*}=A$, *we have*

$$ AA^{\dagger}=A^{\dagger}A=\mathcal{P}_{\operatorname{range}(A)}, $$

*where* $\mathcal{P}_{\operatorname{range}(A)}$ *denotes the orthogonal projection from* $\mathbb{C}^{d}$ *onto* $\operatorname{range}(A)$.

### Lemma 2.11

*For an arbitrary* $s\times t$ *matrix* *A*, *we have*

$$ A^{*}{\mathcal{P}}_{\operatorname{range}(A)}=A^{*}, \qquad A^{\dagger}{\mathcal{P}}_{\operatorname{range}(A)}=A^{\dagger}, $$

$$ \operatorname{range} \bigl(AA^{*} \bigr)=\operatorname{range}(A), \qquad \operatorname{range} \bigl(A^{*}A \bigr)=\operatorname{range} \bigl(A^{*} \bigr), $$

*where* $\mathcal{P}_{\operatorname{range}(A)}$ *denotes the orthogonal projection from* $\mathbb{C}^{s}$ *onto* $\operatorname{range}(A)$.

Let us check the Gabor system $G(\mathbf{g}, \mathbf{a}, \mathbf{b})$ under Assumptions 1 and 2. Since $\mathbb{Z}=\lambda _{l}\mathbb{Z}+\mathbb{N}_{\lambda _{l}}$ for each $1\leq l\leq L$, we have

$$\begin{aligned} \{E_{mb}T_{na_{l}}g_{l}:m, n\in \mathbb{Z}\}&= \{E_{mb}T_{(n\lambda _{l}+\gamma _{l})a_{l}}g_{l}:m,n\in \mathbb{Z}, \gamma _{l}\in \mathbb{N}_{\lambda _{l}}\} \\ &=\{E_{mb}T_{na}g_{l,\gamma _{l}}:m,n\in \mathbb{Z}, \gamma _{l}\in \mathbb{N}_{\lambda _{l}}\}, \end{aligned}$$

where $\lambda _{l}$ and *a* are as in (4) and (5), and $g_{l,\gamma _{l}}=T_{\gamma _{l}a_{l}}g_{l}$. So $G(\mathbf{g}, \mathbf{a}, \mathbf{b})=G(\tilde{\mathbf{g}}, {a}, {b})$, where

$$\begin{aligned}& G(\tilde{\mathbf{g}}, a, b)=\{E_{mb}T_{na}g_{l,\gamma _{l}}: m,n\in \mathbb{Z}, \gamma _{l}\in \mathbb{N}_{\lambda _{l}}, 1\leq l \leq L\}, \\& {\tilde{\mathbf{g}}}=\{g_{1,0}, g_{1,1}, \ldots , g_{1, \lambda _{1}-1}; g_{2,0}, g_{2,1}, \ldots , g_{2, \lambda _{2}-1};\ldots ; g_{L,0}, g_{L,1}, \ldots , g_{L, \lambda _{L}-1}\}. \end{aligned}$$

So the matrix-valued function $G(t,v)$ in Definition 2.1 is exactly ${\Psi }_{\tilde{\mathbf{g}}}(t,v)$ by Definition 2.2 in [31]. It follows that $G(\mathbf{g}, \mathbf{a}, \mathbf{b})$ and $G(\tilde{\mathbf{g}}, a, b)$ have the same frame properties. Therefore, using Theorems 2.9 and 2.14 and Remark 2.10 in [31], we have the following two theorems.

### Theorem 2.1

*For* $\mathbf{g}=\{g_{1}, g_{2}, \ldots , g_{L}\}\subset L^{2}(\mathbb{R})$, *the following are equivalent*:

(i) $G(\mathbf{g}, \mathbf{a}, \mathbf{b})$ *is a frame for* $\mathcal{M}(\mathbf{g}, \mathbf{a}, \mathbf{b})$ *with frame bounds* *A* *and B*.
(ii) $bA G(t,v)G^{*}(t,v)\leq (G(t,v)G^{*}(t,v))^{2}\leq bB G(t,v)G^{*}(t,v)$ *for a*.*e*. $(t,v)\in [0, \frac{1}{bq})\times [0, 1)$.
(iii) $bA G^{*}(t,v)G(t,v)\leq (G^{*}(t,v)G(t,v))^{2}\leq bB G^{*}(t,v)G(t,v)$ *for a*.*e*. $(t,v)\in [0, \frac{1}{bq})\times [0, 1)$.

### Theorem 2.2

*For* $\mathbf{g}=\{g_{1}, g_{2}, \ldots , g_{L}\}\subset L^{2}(\mathbb{R})$, $G(\mathbf{g}, \mathbf{a}, \mathbf{b})$ *is a Riesz basis for* $\mathcal{M}(\mathbf{g}, \mathbf{a}, \mathbf{b})$ *with Riesz bounds* *A* *and* *B* (*an orthonormal basis*) *if and only if*

$$ bAI \leq G(t,v) G^{*}(t,v)\leq bBI \bigl( G(t,v)G^{*}(t,v)=bI \bigr) $$

*for a*.*e*. $(t,v)\in [0, \frac{1}{bq})\times [0, 1)$.

By Lemmas 2.6–2.9, we have following theorem, which characterizes the Gabor duals of type I (resp., type II):

### Theorem 2.3

*Given* $\mathbf{g}=\{g_{1}, g_{2}, \ldots , g_{L}\}\subset L^{2}(\mathbb{R})$, *let* $G(\mathbf{g}, \mathbf{a}, \mathbf{b})$ *be a frame for* $\mathcal{M}(\mathbf{g}, \mathbf{a}, \mathbf{b})$. *Then*, *for an arbitrary* $\mathbf{h}=\{h_{1}, h_{2}, \ldots , h_{L}\}\subset L^{2}(\mathbb{R})$ *with* $G(\mathbf{h}, \mathbf{a}, \mathbf{b})$ *being a Bessel sequence in* $L^{2}(\mathbb{R})$, $G(\mathbf{h}, \mathbf{a}, \mathbf{b})$ *is a Gabor dual of type I* (*type II*) *for* $G(\mathbf{g}, \mathbf{a}, \mathbf{b})$ *if and only if the following hold*:

(i) *there exists a measurable function* $\mathcal{A}:[0, \frac{1}{bq})\times [0, 1)\rightarrow\mathcal{M}_{Q,Q}$ ($\mathcal{B}:[0,\frac{1}{bq})\times [0, 1)\rightarrow \mathcal{M}_{p,p}$) *such that*
  $$ H(t,v)=\mathcal{A}(t,v)G(t,v) \qquad \bigl(H(t,v)= G(t, v)\mathcal{B}(t,v)\bigr); $$
(ii) $G^{*}(t,v)=\frac{1}{b}G^{*}(t,v)H(t,v) G^{*}(t,v)$ *for a*.*e*. $(t,v)\in [0, \frac{1}{bq})\times [0,1)$.

### Theorem 2.4

*Let* $G(\mathbf{g}, \mathbf{a}, \mathbf{b})$ *be a frame for* $\mathcal{M}(\mathbf{g}, \mathbf{a}, \mathbf{b})$. *Then the following are equivalent*:

(i) $G(\mathbf{g}, \mathbf{a}, \mathbf{b})$ *has a unique Gabor dual of type I* (*type II*).
(ii) $\operatorname{rank}(G(t,v))\in \{0, Q\}$ ($\operatorname{rank}(G(t,v))\in \{0, p\}$) *for a*.*e*. $(t,v)\in [0, \frac{1}{bq})\times [0, 1)$.
(iii) $\operatorname{rank}(G(t,v))\in \{0, Q\}$ ($\operatorname{rank}(G(t,v))\in \{0, p\}$) *for a*.*e*. $(t,v)\in \mathbb{R}^{2}$.

### Proof

We only prove “type I” part. The other part can be proved similarly. By Lemma 2.5(ii) we only need to prove the equivalence between (i) and (ii). By Lemmas 2.6 and 2.9, (i) holds if and only if for a function $A:[0, \frac{1}{bq})\times [0, 1)\rightarrow {\mathcal{M}}_{Q, Q}$,

18 $$ G^{*}(t,v)A(t,v)G(t,v)G^{*}(t,v)=0 $$

implies

19 $$ A(t,v)G(t,v)=0 \quad \mbox{for a.e. } \biggl[0, \frac{1}{bq}\biggr)\times [0, 1). $$

Next, we prove the equivalence between (ii) and the above implication. Obviously, (ii) leads to this implication. Next, we prove that the implication fails if (ii) is violated and thus finish the proof. Suppose (ii) does not hold. Then $\operatorname{rank}(G(t,v))\notin \{0, Q\}$ on some subset $E_{0}$ of $[0, \frac{1}{bq})\times [0, 1)$ with $\vert E_{0} \vert >0$. Suppose $\{e_{i}:1\leq i\leq Q\}$ is the orthonormal basis for $\mathbb{C}^{Q}$, where $e_{i}$ is the vector with the *i*th component being 1 and others zeros. Since $\operatorname{rank}(G(t,v))\notin \{0, Q\}$ for $(t,v)\in E_{0}$, there exist $1\leq r\leq Q$ and $E_{1}\subset E_{0}$ with $\vert E_{1} \vert >0$ such that

$$ G^{*}(t,v)e_{r}\neq 0\quad\mbox{and}\quad \operatorname{ker} \bigl(G^{*}(t,v) \bigr)\neq \{0\}\quad \mbox{for }(t,v)\in E_{1}. $$

By the same procedure as in Lemma 4.1 in [31], there exist $1\leq k\leq Q$ and $E_{2}\subset E_{1}$ with $\vert E_{2} \vert >0$ such that $\mathcal{P}(t,v)e_{k}\neq 0$ for $(t,v)\in E_{2}$, where $\mathcal{P}(t,v)$ is the orthogonal projection of $\mathbb{C}^{Q}$ onto $\operatorname{ker}(G^{*}(t,v))$. By the argument of [8], p. 978, $\mathcal{P}(t,v)$ is measurable. Define

$$ A(t,v)= \textstyle\begin{cases} \mathcal{P}(t,v)e_{k}e_{r}^{*} & \mbox{if } (t,v)\in E_{2}, \\ 0 & \mbox{otherwise} \end{cases} $$

for $(t,v)\in [0, \frac{1}{bq})\times [0, 1)$. Then $A(\cdot , \cdot )$ satisfies (18) but does not satisfy (19). The proof is completed. □

### Theorem 2.5

*Given* $\mathbf{g}=\{g_{1}, g_{2}, \ldots , g_{L}\}\subset L^{2}(\mathbb{R})$, *let* $G(\mathbf{g}, \mathbf{a}, \mathbf{b})$ *be a frame for* $\mathcal{M}(\mathbf{g}, \mathbf{a}, \mathbf{b})$. *Then*, *for an arbitrary* $\mathbf{h}=\{h_{1}, h_{2}, \ldots , h_{L}\}\subset L^{2}(\mathbb{R})$ *with* $G(\mathbf{h}, \mathbf{a}, \mathbf{b})$ *being a Bessel sequence in* $L^{2}(\mathbb{R})$, *we have*

(i) $G(\mathbf{h}, \mathbf{a}, \mathbf{b})$ *is a Gabor dual of type I for* $G(\mathbf{g}, \mathbf{a}, \mathbf{b})$ *if and only if there exists a measurable function* $\mathcal{A}:[0, \frac{1}{bq})\times [0, 1)\rightarrow {\mathcal{M}}_{Q, Q}$ *such that*
  20 $$\begin{aligned} H(t,v) &=b \bigl(G(t,v)G^{*}(t,v) \bigr)^{\dagger}G (t,v) \biggl[I-\frac{1}{b}G^{*}(t,v) \mathcal{A}(t,v) G(t,v) \biggr] \\ &\quad {} +\mathcal{A}(t,v)G(t,v) \end{aligned}$$
  *for a*.*e*. $(t,v)\in [0, \frac{1}{bq})\times [0, 1)$.
(ii) $G(\mathbf{h}, \mathbf{a}, \mathbf{b})$ *is a Gabor dual of type II for* $G(\mathbf{g}, \mathbf{a}, \mathbf{b})$ *if there exists a measurable function* $\mathcal{A}:[0, \frac{1}{bq})\times [0, 1)\rightarrow {\mathcal{M}}_{p, p}$ *such that*
  21 $$\begin{aligned} H(t,v)&=bG (t,v) \bigl(G^{*}(t,v)G(t,v) \bigr)^{\dagger} \biggl[I-\frac{1}{b} G^{*}(t,v)G(t,v) \mathcal{A}(t,v) \biggr] \\ &\quad {} +G(t,v)\mathcal{A}(t,v) \end{aligned}$$
  *f or a*.*e*. $(t,v)\in [0, \frac{1}{bq})\times [0, 1)$.
(iii) $G(\mathbf{h}, \mathbf{a}, \mathbf{b})$ *is an oblique dual of* $G(\mathbf{g}, \mathbf{a}, \mathbf{b})$ *if one of the following conditions holds*:
  - $$\begin{aligned} H(t,v)&=bG (t,v) \bigl(G^{*}(t,v)G(t,v) \bigr)^{\dagger} \biggl[I-\frac{1}{b} G^{*}(t,v)\mathcal{A}(t,v)G(t,v) \biggr] \\ &{} +\mathcal{A}(t,v)G(t,v) \end{aligned}$$
    *for some measurable function* $\mathcal{A}:[0, \frac{1}{bq})\times [0, 1)\rightarrow {\mathcal{M}}_{Q, Q}$ *and a*.*e*. $(t,v)\in [0, \frac{1}{bq})\times [0, 1)$;
  - $$\begin{aligned} H(t,v)&=b \bigl(G(t,v)G^{*}(t,v) \bigr)^{\dagger}G (t,v) \biggl[I-\frac{1}{b}G^{*}(t,v)G(t,v)\mathcal{A}(t,v) \biggr] \\ &\quad {} +G(t,v)\mathcal{A}(t,v) \end{aligned}$$
    *for some measurable function* $\mathcal{A}:[0, \frac{1}{bq})\times [0, 1)\rightarrow {\mathcal{M}}_{p, p}$ *and a*.*e*. $(t,v)\in [0, \frac{1}{bq})\times [0, 1)$.

### Proof

We only prove (i); (ii) and (iii) can be proved similarly. First, we assume that (20) holds. Then, applying Lemmas 2.10 and 2.11, we have

$$ \frac{1}{b}G^{*}(t,v)H(t,v) G^{*}(t, v)=G^{*}(t,v), $$

and thus $G(\mathbf{h}, \mathbf{a}, \mathbf{b})$ is a Gabor dual of type I for $G(\mathbf{g}, \mathbf{a}, \mathbf{b})$ by Theorem 2.3.

Now we turn to the converse implication. Suppose $G(\mathbf{h}, \mathbf{a}, \mathbf{b})$ is a Gabor dual of type I for $G(\mathbf{g}, \mathbf{a}, \mathbf{b})$. Then there exists $B(t,v):[0, \frac{1}{bq})\times [0, 1)\rightarrow {\mathcal{M}}_{Q, Q}$ such that

22 $$\begin{aligned} H(t,v)=B(t,v)G(t,v), \qquad G^{*}(t,v)= \frac{1}{b}G^{*} (t,v)H(t,v)G^{*}(t,v) \end{aligned}$$

for a.e. $(t,v)\in [0, \frac{1}{bq})\times [0, 1)$ by Theorem 2.3. It follows that

$$\begin{aligned} &b \bigl(G(t,v)G^{*}(t,v) \bigr)^{\dagger} G(t, v)G^{*}(t,v) \\ &\quad = \bigl(G(t,v)G^{*}(t,v) \bigr)^{\dagger} G(t, v)G^{*}(t,v)H(t,v) G^{*}(t,v) \end{aligned}$$

for a.e. $(t,v)\in [0, \frac{1}{bq})\times [0, 1)$, and thus

23 $$\begin{aligned} b \bigl(G(t,v)G^{*}(t,v) \bigr)^{\dagger} G(t, v)= \bigl(G(t,v)G^{*}(t,v) \bigr)^{\dagger} G(t, v)G^{*}(t,v)H(t,v) \end{aligned}$$

for a.e. $(t,v)\in [0, \frac{1}{bq})\times [0, 1)$ since $\mathbb{C}^{p}=\operatorname{range}(G^{*}(t,v))\oplus { \mathrm {k}er}(G(t,v))$. Put $\mathcal{A}(t,v)=B(t,v)-(G(t,v)G^{*}(t,v))^{\dagger}$. Then (20) holds by Lemmas 2.10 and 2.11 and by a simple computation. The proof is completed. □

## Some examples and remarks

This section is devoted to some examples and remarks. The ideas of this section are borrowed from [1]. Example 2.1 tells us that not every $Q\times p$ matrix-valued function with $L^{2}([0, \frac{1}{bq})\times [0, 1))$-entries determines a $\mathbf{g}=\{g_{1}, g_{2}, \ldots , g_{L}\}\subset L^{2}(\mathbb{R})$ via the Zak transform matrix method if the time shift parameters $a_{1}, a_{2}, \ldots , a_{L}$ are not all the same. Example 3.2 shows that not every subspace mixed Gabor frame $G(\mathbf{g}, \mathbf{a}, \mathbf{b})$ admits an oblique Gabor dual. Therefore, there exist significant differences between mixed multiwindow Gabor frames and usual multiwindow Gabor frames, and there should be many challenging problems in this direction.

### Definition 3.1

Given $\mathbf{g}=\{g_{1}, g_{2}, \ldots , g_{L}\}\subset L^{2}(\mathbb{R})$, let $G(\mathbf{g}, \mathbf{a}, \mathbf{b})$ be a Bessel sequence in $L^{2}(\mathbb{R})$. We say that $G(\mathbf{g}, \mathbf{a}, \mathbf{b})$ has Riesz property if for $c \in l^{2}(\mathbb{Z}^{2}, \mathbb{C}^{L})$, we must have $c=0$ whenever $\mathcal{T}_{\mathbf{g}}c=0$.

By Lemma 2.5(ii) and Theorem 2.1 in [2] we have the following:

### Lemma 3.1

*Given* $\mathbf{g}=\{g_{1}, g_{2}, \ldots , g_{L}\}\subset L^{2}(\mathbb{R})$, *let* $G(\mathbf{g}, \mathbf{a}, \mathbf{b})$ *be a Bessel sequence in* $L^{2}(\mathbb{R})$. *Then* $G(\mathbf{g}, \mathbf{a}, \mathbf{b})$ *has Riesz property if and only if* $\operatorname{rank}(G(t,v))=Q$ *for a*.*e*. $(t,v)\in [0, \frac{1}{bq})\times [0, 1)$.

Next we turn to examples of Theorems 2.1 and 2.2 with $\mathcal{M}(\mathbf{g}, \mathbf{a}, \mathbf{b})\ne L^{2}(\mathbb{R})$. See Examples 4.1, 4.2, 4.5, and 4.6 in [2], for examples with $\mathcal{M}(\mathbf{g}, \mathbf{a}, \mathbf{b})=L^{2}(\mathbb{R})$. Suppose

24 $$\begin{aligned} {\mathbf{a}}=(6, 4)\quad\mbox{and}\quad {\mathbf{b}}= \biggl( \frac{1}{2}, \frac{1}{2} \biggr). \end{aligned}$$

Then $ab=6$ with $p=6$, $q=1$, and $Q=5$. For $\mathbf{g}=\{g_{1}, g_{2}\}$ with $g_{1}$, $g_{2}\in L^{2}(\mathbb{R})$, we associate it with *G* as in Definition 2.1. Then

25 $$\begin{aligned} G(t,v)=\left ( \textstyle\begin{array}{@{}c@{}} G_{1}(t,v) \\ G_{2}(t,v) \end{array}\displaystyle \right ) \end{aligned}$$

with

$$\begin{aligned} G_{1}(t,v)=\left ( \textstyle\begin{array}{@{}c@{}} {\mathcal{G}}_{1}(t,v) \\ \mathcal{G}_{1}(t-6, v) \end{array}\displaystyle \right ) \quad \mbox{and}\quad G_{2}(t,v)=\left ( \textstyle\begin{array}{@{}c@{}} {\mathcal{G}}_{2}(t,v) \\ \mathcal{G}_{2}(t-4, v) \\ \mathcal{G}_{2}(t-8, v) \end{array}\displaystyle \right ) , \end{aligned}$$

where

26 $$\begin{aligned} {\mathcal{G}}_{l}(t,v)= \bigl( \mathcal{Z}_{12}g_{l}(t+2k, v) \bigr) _{0,k} \in {\mathcal{M}}_{1,6} \end{aligned}$$

with $k\in \mathbb{N}_{6}$ and a.e. $(t,v)\in [0, 2)\times [0, 1)$ for $l=1, 2$. By the quasi-periodicity of the Zak transform we have:

$$\begin{aligned}& \mathcal{G}_{1}(t-6, v) = \bigl( e^{2\pi iv}{ \mathcal{Z}}_{12}g_{1}(t+6, v) , e^{2\pi iv}{ \mathcal{Z}}_{12}g_{1}(t+8, v) , e^{2\pi iv}{ \mathcal{Z}}_{12}g_{1}(t+10, v) , \\& \hphantom{\mathcal{G}_{1}(t-6, v) = (}{} \mathcal{Z}_{12}g_{1}(t,v) , \mathcal{Z}_{12}g_{1}(t+2, v) , \mathcal{Z}_{12}g_{1}(t+4, v) \bigr), \\& \mathcal{G}_{2}(t-4, v)= \bigl( e^{2\pi iv}{\mathcal{Z}}_{12}g_{2}(t+8, v) , e^{2\pi iv}{\mathcal{Z}}_{12}g_{2}(t+10, v) , \mathcal{Z}_{12}g_{2}(t,v) , \\& \hphantom{\mathcal{G}_{1}(t-6, v) = (}{} \mathcal{Z}_{12}g_{2}(t+2, v) , \mathcal{Z}_{12}g_{2}(t+4, v) , \mathcal{Z}_{12}g_{2}(t+6, v) \bigr), \\& \mathcal{G}_{2}(t-8, v)= \bigl( e^{2\pi iv}{\mathcal{Z}}_{12}g_{2}(t+4, v) , e^{2\pi iv}{\mathcal{Z}}_{12}g_{2}(t+6, v) , e^{2\pi iv}{\mathcal{Z}}_{12}g_{2}(t+8, v) , \\& \hphantom{\mathcal{G}_{1}(t-6, v) = (}{} \mathcal{Z}_{12}g_{2}(t+10, v) , \mathcal{Z}_{12}g_{2}(t,v) , \mathcal{Z}_{12}g_{2}(t+2, v) \bigr). \end{aligned}$$

Thus, for a.e. $(t,v)\in [0, 2)\times [0, 1)$, $\mathcal{G}_{1}(t-6, v)$ and $\mathcal{G}_{2}(t-4, v)$, $\mathcal{G}_{2}(t-8, v)$ are uniquely determined by $\mathcal{G}_{1}(t,v)$ and $\mathcal{G}_{2}(t,v)$, respectively. Observe that $[0, 2)+2\mathbb{N}_{6}$ is $12\mathbb{Z}$-congruent to $[0, 12)$. It follows that an arbitrary $5\times 6$ matrix-valued function $\mathcal{K}(t,v)$ on $[0, 2)\times [0, 1)$ of the following form determines a unique $\mathbf{g}=\{g_{1}, g_{2}\}$ by

27 $$\begin{aligned}& G(t,v)=\mathcal{K}(t,v)\quad \mbox{for } {\mbox{ a.e. }} (t,v) \in[0, 2]\times [0, 1]: \end{aligned}$$

28 $$\begin{aligned}& {\mathcal{K}}(t,v)= \bigl( \mathcal{K}_{1}(t,v), \mathcal{K}_{2}(t,v) \bigr) \end{aligned}$$

with $\mathcal{K}_{1}(t,v),\mathcal{K}_{2}(t,v)\in {\mathcal{M}}_{5,3}$ and

$$\begin{aligned}& {\mathcal{K}}_{1}(t,v)= \left ( \textstyle\begin{array}{@{}c@{\quad}c@{\quad}c@{}} a(t,v) & \lambda (t,v) a(t,v) & \lambda ^{2}(t,v)a(t,v) \\ 0 & 0 & 0 \\ -\overline{\lambda (t,v) a(t,v)} & \overline{a(t,v)} & 0 \\ 0 & 0 & -\overline{\lambda (t,v) a(t,v)} \\ 0 & 0 & 0 \end{array}\displaystyle \right ) , \\& {\mathcal{K}}_{2}(t,v)=\left ( \textstyle\begin{array}{@{}c@{\quad}c@{\quad}c@{}} 0 & 0 & 0 \\ a(t,v) & \lambda (t,v)a(t,v) & \lambda ^{2}(t,v)a(t,v) \\ 0 & 0 & 0 \\ \overline{a(t,v)} & 0 & 0 \\ 0 & -\overline{\lambda (t,v) a(t,v)} & \overline{a(t,v)} \end{array}\displaystyle \right ) \end{aligned}$$

for a.e. $(t,v)\in [0, 2)\times [0, 1)$.

### Example 3.1

Let **a** and **b** be defined as in (24), and define $\mathbf{g}=\{g_{1}, g_{2}\}$ by (27), where $\lambda (\cdot , \cdot )$ and $a(\cdot , \cdot )$ are continuous on $[0, 2]\times [0, 1]$. Assume that

$$\begin{aligned}& \bigl\vert \lambda (t,v) \bigr\vert < 1, \\& \bigl\vert a(t,v) \bigr\vert ^{2} \bigl(1-2 \bigl\vert \lambda (t,v) \bigr\vert ^{4} -2 \bigl\vert \lambda (t,v) \bigr\vert ^{6}- \bigl\vert \lambda (t,v) \bigr\vert ^{8} \bigr) < 1- \bigl\vert \lambda (t,v) \bigr\vert ^{3}, \\& \bigl\vert a(t,v) \bigr\vert ^{2} \bigl(1+2 \bigl\vert \lambda (t,v) \bigr\vert ^{3} +2 \bigl\vert \lambda (t,v) \bigr\vert ^{5}+ \bigl\vert \lambda (t,v) \bigr\vert ^{7} \bigr) < 1+ \bigl\vert \lambda (t,v) \bigr\vert ^{3} \end{aligned}$$

for $(t,v)\in [0, 2]\times [0, 1]$ satisfying $a(t,v)\neq 0$. Then $G(\mathbf{g}, \mathbf{a}, \mathbf{b})$ is a frame for $\mathcal{M}(\mathbf{g}, \mathbf{a}, \mathbf{b})$, and $\mathcal{M}(\mathbf{g}, \mathbf{a}, \mathbf{b})\ne L^{2}(\mathbb{R})$. In particular, $G(\mathbf{g}, \mathbf{a}, \mathbf{b})$ is a Riesz basis for $\mathcal{M}(\mathbf{g}, \mathbf{a}, \mathbf{b})$ if and only if, in addition,

29 $$\begin{aligned} a(t,v)\lambda (t,v)\neq 0\quad \mbox{for a.e. } (t,v)\in [0, 2) \times [0, 1). \end{aligned}$$

### Proof

Since $G(t,v)$ is a $5\times 6$ matrix, its rank cannot be $p=6$. So $\mathcal{M}(\mathbf{g}, \mathbf{a}, \mathbf{b})\ne L^{2}(\mathbb{R})$ by Lemma 2.4. Next, we prove that $G(\mathbf{g}, \mathbf{a}, \mathbf{b})$ is a frame for $\mathcal{M}(\mathbf{g}, \mathbf{a}, \mathbf{b})$. By a simple computation we have

30 $$\begin{aligned}& \bigl\langle \bigl(G(t,v)G^{*}(t,v) \bigr)^{2}x, x \bigr\rangle = \bigl\vert a(t,v) \bigr\vert ^{2}\sum_{l=1}^{5} \alpha _{l}(t,v)\vert x_{l} \vert ^{2}+\beta (t, v), \end{aligned}$$

31 $$\begin{aligned}& \bigl\langle G(t,v)G^{*}(t,v)x, x \bigr\rangle = \bigl\vert a(t,v) \bigr\vert ^{2}\sum_{l=1}^{5} \tilde{\alpha }_{l}(t,v)\vert x_{l} \vert ^{2} +\tilde{\beta }(t,v) \end{aligned}$$

for $(t,v)\in [0, 2]\times [0, 1]$ and $x\in \mathbb{C}^{5}$, where

$$\begin{aligned}& \alpha _{1}(t,v)= \bigl\vert \lambda (t,v) \bigr\vert ^{3}+ \bigl(1+ \bigl\vert \lambda (t,v) \bigr\vert ^{2}+ \bigl\vert \lambda (t,v) \bigr\vert ^{4} \bigr)^{2} \bigl\vert a(t,v) \bigr\vert ^{2}, \\& \alpha _{2}(t,v)=1+ \bigl(1+ \bigl\vert \lambda (t,v) \bigr\vert ^{2}+ \bigl\vert \lambda (t,v) \bigr\vert ^{4} \bigr)^{2} \bigl\vert a(t,v) \bigr\vert ^{2}, \\& \alpha _{4}(t,v)=1+ \bigl\vert \lambda (t,v) \bigr\vert ^{3}+ \bigl(1+ \bigl\vert \lambda (t,v) \bigr\vert ^{2} \bigr)^{2} \bigl\vert a(t,v) \bigr\vert ^{2}, \\& \alpha _{3}(t,v)=\alpha _{5}(t,v) = \bigl(1+ \bigl\vert \lambda (t,v) \bigr\vert ^{2} \bigr)^{2} \bigl\vert a(t,v) \bigr\vert ^{2}, \\& \beta (t,v) = 2\operatorname{Re} \bigl( \bigl[\lambda (t,v)^{3} \bigl(2+2\bigl\vert \lambda (t,v) \bigr\vert ^{2} +\bigl\vert \lambda (t,v) \bigr\vert ^{4}\bigr)a(t,v)^{2}\bigl\vert a(t,v) \bigr\vert ^{2}\bigr] \bar{x}_{1}x_{4} \bigr) \\& \hphantom{\beta (t,v) = }{} +2\operatorname{Re} \bigl( \bigl[\bigl(2+2\bigl\vert \lambda (t,v) \bigr\vert ^{2} +\bigl\vert \lambda (t,v) \bigr\vert ^{4}\bigr)a(t,v)^{2}\bigl\vert a(t,v) \bigr\vert ^{2}\bigr] \bar{x}_{2}x_{4} \bigr) \\& \hphantom{\beta (t,v) = }{} -2\operatorname{Re} \bigl( \lambda (t,v)^{3} \bigl\vert a(t,v) \bigr\vert ^{2}\bar{x}_{1}x_{2} \bigr) , \\& \tilde{\alpha }_{1}(t,v)=\tilde{\alpha }_{2}(t,v) =1+ \bigl\vert \lambda (t,v) \bigr\vert ^{2}+ \bigl\vert \lambda (t, v) \bigr\vert ^{4}, \\& \tilde{\alpha }_{3}(t,v)=\tilde{\alpha }_{4}(t,v) = \tilde{\alpha }_{5}(t,v) =1+ \bigl\vert \lambda (t,v) \bigr\vert ^{2}, \\& \tilde{\beta }(t,v)=-2\operatorname{Re} \bigl(\lambda (t,v)^{3}a(t, v)^{2} \bar{x}_{1}x_{4} \bigr) +2 \operatorname{Re} \bigl(a(t,v)^{2} \bar{x}_{2}x_{4} \bigr). \end{aligned}$$

It is easy to check that

$$ \bigl\vert \beta (t,v) \bigr\vert \leq \bigl\vert a(t,v) \bigr\vert ^{2} \bigl[ \beta _{1}(t,v)\vert x_{1} \vert ^{2} +\beta _{2}(t,v)\vert x_{2} \vert ^{2}+\beta _{4}(t,v)\vert x_{4} \vert ^{2} \bigr] , $$

w here

$$\begin{aligned}& \beta _{1}(t,v) = \bigl\vert \lambda (t,v) \bigr\vert ^{3} + \bigl\vert a(t,v) \bigr\vert ^{2} \bigl(2 \bigl\vert \lambda (t,v) \bigr\vert ^{3} +2 \bigl\vert \lambda (t, v) \bigr\vert ^{5}+ \bigl\vert \lambda (t,v) \bigr\vert ^{7} \bigr), \\& \beta _{2}(t,v) = \bigl\vert \lambda (t,v) \bigr\vert ^{3} + \bigl\vert a(t,v) \bigr\vert ^{2} \bigl(2+2 \bigl\vert \lambda (t,v) \bigr\vert ^{2}+ \bigl\vert \lambda (t,v) \bigr\vert ^{4} \bigr), \\& \beta _{4}(t,v) = \bigl\vert a(t,v) \bigr\vert ^{2} \bigl( 2+2 \bigl\vert \lambda (t,v) \bigr\vert ^{2} +2 \bigl\vert \lambda (t,v) \bigr\vert ^{3} + \bigl\vert \lambda (t,v) \bigr\vert ^{4} \bigr) \\& \hphantom{\beta_{4} (t,v) = }{}+ \bigl\vert a(t,v) \bigr\vert ^{2} \bigl(2 \bigl\vert \lambda (t,v) \bigr\vert ^{5}+ \bigl\vert \lambda (t,v) \bigr\vert ^{7} \bigr). \end{aligned}$$

We have

$$\begin{aligned} \bigl\vert \tilde{\beta }(t,v) \bigr\vert \leq \bigl\vert a(t,v) \bigr\vert ^{2} \bigl[ \tilde{\beta }_{1}(t,v)\vert x_{1} \vert ^{2} +\tilde{\beta }_{2}(t,v) \vert x_{2} \vert ^{2} +\tilde{\beta }_{4}(t, v)\vert x_{4} \vert ^{2} \bigr] , \end{aligned}$$

where

$$\begin{aligned} \tilde{\beta }_{1}(t,v)= \bigl\vert \lambda (t,v) \bigr\vert ^{3},\qquad \tilde{\beta }_{2}(t,v)= 1, \quad \quad \tilde{ \beta }_{4}(t,v)=1+ \bigl\vert \lambda (t,v) \bigr\vert ^{3}. \end{aligned}$$

Write

$$\begin{aligned}& C_{l}(t,v)= \alpha _{l}(t,v)+\beta _{l}(t, v), \qquad \widetilde{C}_{l}(t,v)= {\alpha }_{l}(t, v)-{\beta }_{l}(t,v)\quad \mbox{for }l=1, 2, 4, \\& C(t,v)= \bigl(1+ \bigl\vert \lambda (t,v) \bigr\vert ^{2} \bigr)^{2} \bigl\vert a(t,v) \bigr\vert ^{2}, \\& D_{l}(t,v)= \tilde{\alpha }_{l}(t,v)+\tilde{\beta }_{l}(t,v),\qquad \widetilde{D}_{l}(t,v)= \tilde{ \alpha }_{l}(t,v)-\tilde{\beta }_{l}(t,v)\quad \mbox{for }l=1, 2, 4, \\& D(t,v)= \bigl\vert \lambda (t,v) \bigr\vert ^{2}+1. \end{aligned}$$

Take

$$\begin{aligned}& \textstyle\begin{cases} \tilde{C}=\min \{ C(t,v), \tilde{C}_{l}(t,v):l=1, 2, 4, (t,v)\in [0, 2]\times [0, 1] \}, \\ {C}=\max \{ C(t,v), {C}_{l}(t,v):l=1, 2, 4, (t,v)\in [0, 2]\times [0, 1] \} , \end{cases}\displaystyle \\& \textstyle\begin{cases} \tilde{D}=\min \{ D(t,v), \tilde{D}_{l}(t,v):l=1, 2, 4, (t,v)\in [0, 2]\times [0, 1] \} , \\ {D}=\max \{ D(t,v), {D}_{l}(t,v):l=1, 2, 4, (t,v)\in [0, 2]\times [0, 1] \} . \end{cases}\displaystyle \end{aligned}$$

Then

$$\begin{aligned}& \tilde{C} \bigl\vert a(t,v) \bigr\vert ^{2}\Vert x \Vert ^{2}\le \bigl\langle \bigl(G(t,v)G^{*}(t,v) \bigr)^{2}x, x \bigr\rangle \leq C \bigl\vert a(t,v) \bigr\vert ^{2}\Vert x \Vert ^{2}, \\& \widetilde{D}\bigl\vert a(t,v) \bigr\vert ^{2}\Vert x \Vert ^{2}\le \bigl\langle G(t,v)G^{*}(t,v)x, x\bigr\rangle \leq D \bigl\vert a(t,v) \bigr\vert ^{2}\Vert x \Vert ^{2}. \end{aligned}$$

It follows that

$$ \frac{1}{2}A \bigl\langle G(t,v)G^{*}(t,v)x, x \bigr\rangle \leq \bigl\langle \bigl(G(t,v)G^{*}(t,v) \bigr)^{2}x, x \bigr\rangle \leq \frac{1}{2}B \bigl\langle G(t,v)G^{*}(t,v)x, x \bigr\rangle $$

for $x\in \mathbb{C}^{5}$and a.e. $(t,v)\in [0, 2)\times [0, 1)$, and thus

32 $$\begin{aligned} \frac{1}{2}A G(t,v)G^{*}(t,v) \le \bigl(G(t, v)G^{*}(t,v) \bigr)^{2}\le \frac{1}{2}B G(t, v)G^{*}(t,v) \end{aligned}$$

for a.e. $(t,v)\in [0, 2)\times [0, 1)$, where $A=\frac{2\widetilde{C}}{D}$, $B=\frac{2C}{\widetilde{D}}$. By Theorem 2.1, $G(\mathbf{g}, \mathbf{a}, \mathbf{b})$ is a frame for $\mathcal{M}(\mathbf{g}, \mathbf{a}, \mathbf{b})$ with frame bounds *A* and *B*.

By simple computation, (29) holds if and only if $\operatorname{rank}G(t,v)=5$ for a.e. $(t,v)\in [0, 2)\times [0, 1)$ or, equivalently, $G(\mathbf{g}, \mathbf{a}, \mathbf{b})$ is a Riesz basis for $\mathcal{M}(\mathbf{g}, \mathbf{a}, \mathbf{b})$ by Lemma 3.1. □

### Remark 3.1

Not every subspace mixed Gabor frame $G(\mathbf{g}, \mathbf{a}, \mathbf{b})$ admits an oblique Gabor dual.

We show it by revisiting Example 3.1. Let us make the additional assumption that $\lambda (t,v)=0$ and $a(t,v)\ne 0$ for a.e. $(t,v)\in [0, 2)\times [0, 1]$. Then $G(\mathbf{g}, \mathbf{a}, \mathbf{b})$ is a frame but not a Riesz basis for $\mathcal{M}(\mathbf{g}, \mathbf{a}, \mathbf{b})$ by Example 3.1. Suppose $G(\mathbf{h}, \mathbf{a}, \mathbf{b})$ with $\mathbf{h}=\{h_{1}, h_{2}\}$ is an oblique Gabor dual for $G(\mathbf{g}, \mathbf{a}, \mathbf{b})$. Then

33 $$ G^{*}(t,v)=2G^{*}(t,v)H(t,v) G^{*}(t,v)\quad \mbox{for a.e. } (t,v)\in [0, 2)\times [0, 1) $$

by Lemma 2.9. Writing out $(0, 0)$-, $(3, 1)$-, and $(5, 4)$-entries of both sides, we have

34 $$\begin{aligned} & 2 \bigl( \overline{a(t,v)} \bigr) ^{2}{ \mathcal{Z}}_{12}h_{1}(t,v)=\overline{a(t,v)}, \end{aligned}$$

35 $$\begin{aligned} & 2 \bigl( \overline{a(t,v)} \bigr) ^{2}{ \mathcal{Z}}_{12}h_{1}(t,v)+2 \bigl\vert a(t,v) \bigr\vert ^{2} {\mathcal{Z}}_{12}h_{2}(t+2, v)= \overline{a(t,v)}, \end{aligned}$$

36 $$\begin{aligned} & 2 \bigl( a(t,v) \bigr) ^{2}{\mathcal{Z}}_{12}h_{2}(t+2, v)=a(t,v) \end{aligned}$$

for a.e. $(t,v)\in [0, 2)\times [0, 1)$. By (35) and (36) we have $2\overline{a(t,v)} {\mathcal{Z}}_{12}h_{1}(t,v)=0 $ for a.e. $(t,v)\in [0, 2)\times [0, 1)$. This contradicts (34).

Observe that $a_{1}\ne a_{2}$ in Remark 3.1 ($a_{1}=6$ and $a_{2}=4$). It is natural to ask:

Does every subspace Gabor frame $G(\mathbf{g}, \mathbf{a}, \mathbf{b})$ admit no oblique Gabor dual whenever $a_{l}$, $1\le l\le L$, are not all the same?

The following example gives a negative answer to this question.

### Example 3.2

Let $\mathbf{a}=(1, 2)$ and $\mathbf{b}=(\frac{1}{3}, \frac{1}{3})$. Assume that

$$ \mathcal{J}(t,v)=\left ( \textstyle\begin{array}{@{}c@{}} {\mathcal{J}}_{1}(t,v) \\ \mathcal{J}_{2}(t,v) \end{array}\displaystyle \right ) \quad \mbox{and}\quad {\mathcal{E}}(t,v)=\left ( \textstyle\begin{array}{@{}c@{}} {\mathcal{E}}_{1}(t,v)\\ \mathcal{E}_{2}(t,v) \end{array}\displaystyle \right ) $$

have the form

$$\begin{aligned}& {\mathcal{J}}_{1}(t,v)=\left ( \textstyle\begin{array}{@{}c@{\quad}c@{}} a_{0,0}(t,v) & a_{0,1}(t,v) \\ a_{1,0}(t,v) & a_{1,1}(t,v) \\ a_{2,0}(t,v) & a_{2,1}(t,v) \\ a_{2,1}(t,v) & e^{-2\pi i v}a_{2,0}(t,v) \\ e^{2\pi i v}a_{0,1}(t,v) &a_{0,0}(t,v) \\ e^{2\pi i v}a_{1,1}(t,v) &a_{1,0}(t,v) \end{array}\displaystyle \right ) , \\& {\mathcal{E}}_{1}(t,v)=\left ( \textstyle\begin{array}{@{}c@{\quad}c@{}} c_{0,0}(t,v) & c_{0,1}(t,v) \\ c_{1,0}(t,v) & c_{1,1}(t,v) \\ c_{2,0}(t,v) & c_{2,1}(t,v) \\ c_{2,1}(t,v) & e^{-2\pi i v}c_{2,0}(t,v) \\ e^{2\pi i v}c_{0,1}(t,v) &c_{0,0}(t,v) \\ e^{2\pi i v}c_{1,1}(t,v) &c_{1,0}(t,v) \end{array}\displaystyle \right ) , \end{aligned}$$

and

$$ {\mathcal{J}}_{2}(t,v)=\left ( \textstyle\begin{array}{@{}c@{\quad}c@{}} b_{0,0}(t,v) & b_{0,1}(t,v) \\ b_{1,0}(t,v) & b_{1,1}(t,v) \\ b_{2,0}(t,v) & b_{2,1}(t,v) \end{array}\displaystyle \right ) ,\qquad { \mathcal{E}}_{2}(t,v)=\left ( \textstyle\begin{array}{@{}c@{\quad}c@{}} d_{0,0}(t,v) & d_{0,1}(t,v) \\ d_{1,0}(t,v) & d_{1,1}(t,v) \\ d_{2,0}(t,v) & d_{2,1}(t,v) \end{array}\displaystyle \right ) $$

for a.e. $(t,v)\in [0, 2)\times [0, 1)$ with all entries of $\mathcal{J}(t,v)$ and $\mathcal{E}(t,v)$ in $L^{\infty }([0, 2)\times [0, 1))$, that $(\mathcal{J}_{1}(t,v))^{*}{\mathcal{E}}_{1}(t,v)$ has the form

$$ \left ( \textstyle\begin{array}{@{}c@{\quad}c@{}} A(t,v) & 0 \\ 0 & A(t,v) \end{array}\displaystyle \right ) $$

and $(\mathcal{J}(t,v))^{*}{\mathcal{J}}(t,v)$ has the form

$$ \left ( \textstyle\begin{array}{@{}c@{\quad}c@{}} B(t,v) & 0 \\ 0 & B(t,v) \end{array}\displaystyle \right ) $$

for $(t,v)\in [0, 2)\times [0, 1)$, and that

$$ \bigl(\mathcal{J}_{2}(t,v) \bigr)^{*}{\mathcal{E}}_{2}(t, v)= \left ( \textstyle\begin{array}{@{}c@{\quad}c@{}} \frac{1}{3}-A(t,v) & 0 \\ 0 & \frac{1}{3}-A(t,v) \end{array}\displaystyle \right ) $$

for $(t,v)\in [0, 2)\times [0, 1)$ satisfying $B(t,v)\ne 0$. Define $\mathbf{g}=\{g_{1}, g_{2}\}$ and $\mathbf{h}=\{h_{1}, h_{2}\}$ by

$$ G(t,v)=\mathcal{J}(t,v)\quad\mbox{and}\quad H(t,v)=\mathcal{E}(t,v) \quad \mbox{for } (t,v)\in [0, 2)\times [0, 1). $$

Then **g** and **h** are well defined by the quasi-periodicity of the Zak transform $\mathcal{Z}_{6}$ and $6\mathbb{Z}$-congruence between $[0, 1)-2\mathbb{N}_{3}+3\mathbb{N}_{2}$ and $[0, 6)$, and $G(\mathbf{g}, \mathbf{a}, \mathbf{b})$ and $G(\mathbf{h}, \mathbf{a}, \mathbf{b})$ are both Bessel sequences by the quasi-periodicity of the Zak transform and Lemma 2.3(vi). A simple computation shows that

$$ G^{*}(t,v)G(t,v) =3G^{*}(t,v)H(t,v) G^{*}(t,v)G(t,v) $$

for $(t,v)\in [0, 2)\times [0, 1)$. This implies that $G^{*}(t,v) =3G^{*}(t,v)H(t,v) G^{*}(t,v)$ for $(t,v)\in [0, 2)\times [0, 1)$ since $\mathbb{C}^{9}=\operatorname{range}(G(t,v))\oplus {\mathrm{{ker}}}(G^{*}(t,v))$. So $G(\mathbf{h}, \mathbf{a}, \mathbf{b})$ is an oblique Gabor dual for $G(\mathbf{h}, \mathbf{a}, \mathbf{b})$ by Lemma 2.9.

## Conclusions

A mixed multiwindow Gabor system is one of generalizations of multiwindow Gabor systems, whose time-frequency shifts vary with the windows. This paper addresses subspace mixed multiwindow Gabor systems with rational time-frequency product lattices. Using a suitable Zak-transform matrix method, in this paper, we characterize subspace mixed multiwindow Gabor frames and their Gabor duals, obtain explicit expressions of Gabor duals, and characterize the uniqueness of Gabor duals. Some provided examples show that there exist significant differences between mixed multiwindow Gabor frames and usual multiwindow Gabor frames.

## References

[1] Li Y.-Z., Zhang Y. (2013). Discrete subspace multiwindow Gabor frames and their duals. Abstr. Appl. Anal..

[2] Zhang Y., Li Y.-Z. (2014). Characterization of rational time-frequency multi-window Gabor frames and their duals. J. Korean Math. Soc..

[3] Christensen O. (2003). An Introduction to Frames and Riesz Bases.

[4] Feichtinger H.G., Strohmer T. (1998). Gabor Analysis and Algorithms, Theory and Applications.

[5] Feichtinger H.G., Strohmer T. (2003). Advances in Gabor Analysis.

[6] Young R.M. (1980). An Introduction to Nonharmonic Fourier Series.

[7] Christiansen L.H. (2015). Pairs of dual Gabor frames generated by functions of Hilbert–Schmidt type. Adv. Comput. Math..

[8] Daubechies I. (1990). The wavelet transform, time-frequency localization and signal analysis. IEEE Trans. Inf. Theory.

[9] Gröchenig K. (2001). Foundations of Time-Frequency Analysis.

[10] Zibulski M., Zeevi Y.Y. (1997). Analysis of multiwindow Gabor-type schemes by frame methods. Appl. Comput. Harmon. Anal..

[11] Zeevi Y.Y., Zibulski M., Porat M. (1998). Multi-window Gabor schemes in signal and image representations. Gabor Analysis and Algorithms.

[12] Zeevi Y.Y. (2001). Multiwindow Gabor-type representations and signal representation by partial information. Twentieth Century Harmonic Analysis – a Celebration (Il Ciocco, 2000).

[13] Akinlar M.A., Gabardo J.-P. (2008). Oblique duals associated with rational subspace Gabor frames. J. Integral Equ. Appl..

[14] Gabardo J.-P., Han D. (2004). Balian–Low phenomenon for subspace Gabor frames. J. Math. Phys..

[15] Heil C. (2007). History and evolution of the density theorem for Gabor frames. J. Fourier Anal. Appl..

[16] Jakobsen M.S., Lemvig J. (2016). Density and duality theorems for regular Gabor frames. J. Funct. Anal..

[17] Balan R., Christensen J.G., Krishtal I.A., Okoudjou K.A., Romero J.L. (2014). Multi-window Gabor frames in amalgam spaces. Math. Res. Lett..

[18] Feichtinger H.G., Onchiş D.M. (2010). Constructive reconstruction from irregular sampling in multi-window spline-type spaces. Progress in Analysis and Its Applications.

[19] Feichtinger H.G., Onchis D.M. (2010). Constructive realization of dual systems for generators of multi-window spline-type spaces. J. Comput. Appl. Math..

[20] Jaillet F., Torrésani B. (2007). Time-frequency jigsaw puzzle: adaptive multiwindow and multilayered Gabor expansions. Int. J. Wavelets Multiresolut. Inf. Process..

[21] Casazza P.G., Christensen O. (2001). Weyl–Heisenberg frames for subspaces of $L^{2}(\mathbb{R})$. Proc. Am. Math. Soc..

[22] Gabardo J.-P., Han D. (2001). Subspace Weyl–Heisenberg frames. J. Fourier Anal. Appl..

[23] Gabardo J.-P., Han D. (2004). The uniqueness of the dual of Weyl–Heisenberg subspace frames. Appl. Comput. Harmon. Anal..

[24] Gabardo J.-P., Li Y.-Z. (2009). Density results for Gabor systems associated with periodic subsets of the real line. J. Approx. Theory.

[25] Gabardo J.P., Li Y.-Z. (2014). Rational time-frequency Gabor frames associated with periodic subsets of the real line. Int. J. Wavelets Multiresolut. Inf. Process..

[26] Lian Q.-F., Li Y.-Z. (2011). Gabor frame sets for subspaces. Adv. Comput. Math..

[27] Li Y.-Z., Jia H.-F. (2015). Weak Gabor bi-frames on periodic subsets of the real line. Int. J. Wavelets Multiresolut. Inf. Process..

[28] Li Y.-Z., Lian Q.-F. (2009). Gabor systems on discrete periodic sets. Sci. China Ser. A.

[29] Lian Q.-F., Li Y.-Z. (2009). The duals of Gabor frames on discrete periodic sets. J. Math. Phys..

[30] Gabardo J.-P., Han D., Li Y.-Z. (2013). Lattice tiling and density conditions for subspace Gabor frames. J. Funct. Anal..

[31] Zhang Y., Li Y.-Z. (2014). Rational time-frequency multi-window subspace Gabor frames and their Gabor duals. Sci. China Math..

